# A comprehensive breath test that confirms recent use of inhaled cannabis within the impairment window

**DOI:** 10.1038/s41598-021-02137-x

**Published:** 2021-11-23

**Authors:** Michael W. DeGregorio, Gregory T. Wurz, Edward Montoya, Chiao-Jung Kao

**Affiliations:** 1 RCU Labs, Inc., 408 Sunrise Avenue, Roseville, CA 95661-4123 USA; 2 Cancer Immunotherapy Research Institute, 408 Sunrise Avenue, Roseville, CA 95661 USA

**Keywords:** Drug regulation, Pharmacodynamics, Translational research

## Abstract

Legalization of cannabis for medicinal and/or recreational use is expanding globally. Although cannabis is being regulated country by country, an accurate recent use test with indisputable results correlated with impairment has yet to be discovered. In the present study, a new approach for determining recent cannabis use within the impairment window after smoking was developed by studying 74 subjects with a mean age of 25 years and average use history of 9 years. Horizontal gaze nystagmus was evaluated along with subject self-assessments of impairment, and blood and breath samples were collected before and after smoking cannabis. Breath and blood pharmacokinetic parameters and cannabinoid profiles determined recent use within the impairment window. No subjects were positive for recent use pre-smoking, although all subjects had detectable cannabinoids in breath samples. We describe an inhaled cannabis recent use test that correlates with impairment and helps protect against wrongful prosecution and workplace discrimination.

## Introduction

Despite several different available testing methods for assessing cannabis use, the ability to effectively establish recent use within the impairment window, generally accepted to be approximately 3 h^1,2^, has remained elusive. As a result, inappropriate interpretation of test results by employers, for example, can lead to job applicants not getting hired, or to the wrongful termination of current, non-federal employees who are using cannabis lawfully and responsibly in jurisdictions that allow such use, and where employees are not serving in safety-sensitive positions. As of July 2021, recreational cannabis has been legalized in 19 U.S. states plus Washington, D.C. Some of these states, including Nevada, New Jersey, and New York, have begun to enact laws that ban employment discrimination against current or prospective employees who engage in legal, off-duty recreational cannabis use. Ironically, Colorado, one of the first states to legalize recreational cannabis, provides little legal protection of off-duty cannabis use by employees^3^. At the same time, studies have suggested that the legalization of recreational cannabis has been associated with an increase in fatal traffic collisions^4,5^. Based on data collected from 2007 to 2018, a recently published study showed that in U.S. states where recreational cannabis is legal, there has been a 15% increase in fatal collisions and a 16% increase in associated deaths, changes that were sustained beyond the first year following legalization^6^. Clearly, a new type of test that can identify recent cannabis use within the impairment window is needed to help bring an end to cannabis discrimination practices in the workplace while also accurately detecting inappropriate cannabis use, e.g., driving under the influence (DUI), to protect public safety.

While it has been known for over 30 years that ∆^9^-tetrahydrocannabinol (∆^9^-THC) can be detected in exhaled breath^7^, only relatively recently has this matrix been explored as a potential means of establishing recent cannabis use within the impairment window^8,9^. Exhaled breath testing for recent use is predicated on a short period of detection for ∆^9^-THC within the impairment window. A study by Himes et al. suggested that ∆^9^-THC is generally detectable in breath for only about two hours after smoking even in chronic users^8^, but more recent studies have shown that ∆^9^-THC remains detectable in breath up to several days since last use^10,11^. This is a major finding because no meaningful correlation has yet been established between impairment and ∆^9^-THC levels in any matrix tested to date. Because the leading technologies for breath-based testing for recent cannabis use, such as described by Lynch et al.^10^ and others^12^, rely solely on the detection of ∆^9^-THC, there is a real potential for false positive test results due to the presence of ∆^9^-THC in breath outside of the impairment window.

The complex nature of cannabinoid pharmacokinetics and pharmacodynamics calls for a new approach for effectively determining recent use of cannabis within the impairment window. We hypothesized that incorporating both exhaled breath and blood testing could improve the accuracy of current breath-based testing methods for recent cannabis use, prevent false positive test results, and definitively establish whether a subject is in the impairment window following the use of cannabis through inhalation. We sought to develop a test that incorporates pharmacological changes in ∆^9^-THC and other cannabinoids in breath over time after smoking, which is the most common route of cannabis administration^13^. This approach requires collection of two breath samples separated by a known time interval, which is a critical difference compared to current testing methods that rely on a single sample, as well as a blood sample. The two-sample strategy makes detection of ∆^9^-THC and other cannabinoids possible in their distribution phases, which occur during the first few hours after smoking, and for the evaluation of how cannabinoid levels in breath are changing with time relative to blood. Liquid chromatography high-resolution mass spectrometry (LC-HRMS) bioanalytical methods for the quantification of ∆^9^-THC and other cannabinoids in exhaled breath and whole blood^14^ were developed and validated for this purpose.

Here we describe the clinical development of a comprehensive breath and blood-based test and its application in determining recent cannabis use and impairment after smoking.

## Results

### Clinical study: subject demographics

A total of 74 subjects were recruited over a one-year period. The majority of subjects were recreational cannabis users, with some reporting both recreational and medicinal use. For most subjects, smoking and/or vaping was the primary route of use, while some subjects also reported use of cannabis edibles. When asked about frequency of use, most subjects reported daily use (14 out of the last 14 days). There was an approximate 3:1 ratio of males (56) to females (18), and the subjects ranged in age from 21 to 42 years, with an average age of 25.0 ± 4.5 years and a median age of 23 years (see Supplementary Table S1). The subjects reported a mean cannabis use history of 9.0 ± 4.4 years.

### Impairment window following cannabis smoking

**Figure 1:**
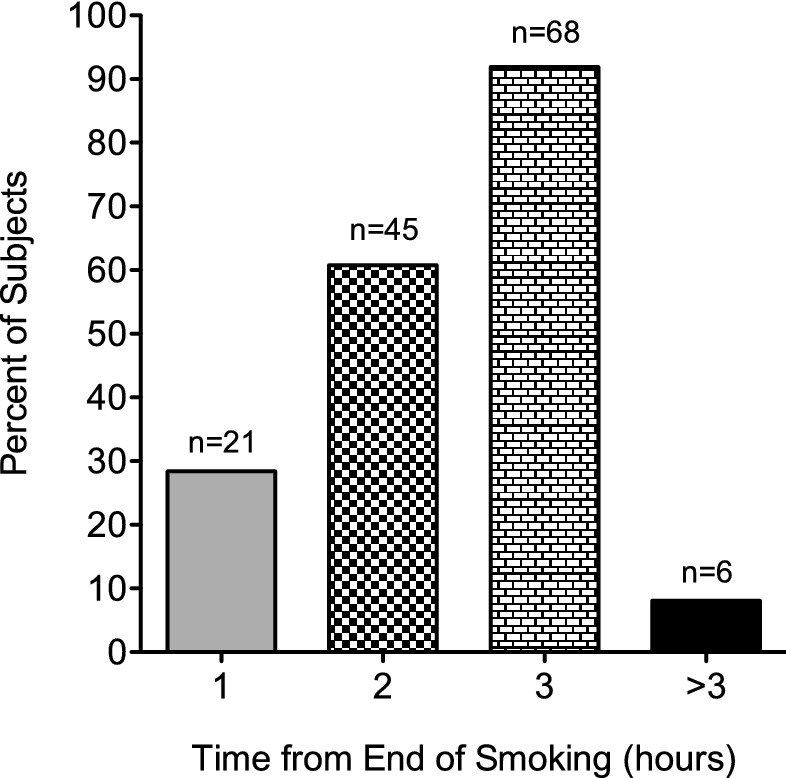
Impairment windows following cannabis smoking. The window of impairment after smoking cannabis was evaluated in 74 subjects who self-assessed their level of impairment prior to smoking and at various time points post-smoking using a 10-point scale.

The window of impairment following cannabis smoking was evaluated in 74 subjects who self-assessed their impairment level prior to smoking and at various time points post-smoking using a 10-point scale, where zero denoted no impairment and 10 denoted maximal impairment (incapacitation) for that individual. Some subjects did not finish smoking their cannabis cigarettes because they considered themselves completely impaired; however, all of the subjects experienced some impairment to the level where they felt they could no longer drive, which was the desired effect, but they were not maximally impaired. Among these subjects, all 74 (100%) reported peak impairment within the first hour after smoking (48 subjects immediately after smoking, 24 subjects at 20 min post-smoking, and two subjects at 60 min post-smoking) (see Supplementary Table S2). The overall window of impairment was found to be approximately three hours after smoking, at which time 68 of 74 subjects (92%) last reported any impairment. There were six subjects (8%) still reporting some degree of impairment outside this window (see Fig. 1). In these six subjects, the percent maximum impairment was an average 23.0 ± 21.7% (range 11.1 to 66.7%). To normalize self-assessed impairment data, the reported impairment levels at each time point for each subject (Supplementary Table S2) were divided by the maximum reported impairment level for each subject, with the result expressed as a percentage, as shown in Table 1.

**Table 1:** Percent maximum self-assessed impairment.

Subject	Percent maximum self-assessed impairment post-smoking (minutes)										
	Pre-smoking	0	20	40	60	80	120	140	180	200	240
1	0.0	66.7	100.0	–*	83.3	50.0	16.7	0.0	0.0	0.0	–
2	0.0	33.3	100.0	–	66.7	66.7	0.0	0.0	0.0	0.0	–
3	0.0	66.7	83.3	–	100.0	100.0	83.3	66.7	33.3	0.0	–
4	0.0	100.0	84.6	–	46.2	23.1	7.7	7.7	0.0	0.0	–
5	0.0	60.0	100.0	–	60.0	20.0	0.0	0.0	0.0	0.0	–
6	0.0	100.0	62.5	–	12.5	0.0	0.0	0.0	0.0	0.0	–
7	0.0	100.0	83.3	–	66.7	66.7	50.0	33.3	16.7	0.0	–
8	0.0	66.7	100.0	–	66.7	66.7	33.3	0.0	0.0	0.0	–
9	0.0	100.0	80.0	–	40.0	0.0	0.0	0.0	0.0	0.0	–
10	0.0	100.0	62.5	–	25.0	12.5	0.0	0.0	0.0	0.0	–
11	0.0	100.0	100.0	–	66.7	50.0	33.3	0.0	0.0	0.0	–
12	0.0	100.0	90.0	–	80.0	80.0	60.0	50.0	30.0	20.0	–
13	0.0	100.0	80.0	–	0.0	0.0	0.0	0.0	0.0	0.0	–
14	0.0	100.0	83.3	–	16.7	16.7	16.7	0.0	0.0	0.0	–
15	0.0	100.0	83.3	–	50.0	33.3	0.0	0.0	0.0	0.0	–
16	0.0	100.0	66.7	–	33.3	33.3	0.0	0.0	0.0	0.0	–
17	0.0	75.0	100.0	–	25.0	0.0	0.0	0.0	0.0	0.0	–
18	0.0	87.5	100.0	–	50.0	50.0	37.5	12.5	12.5	0.0	–
19	0.0	66.7	100.0	–	55.6	44.4	22.2	11.1	0.0	0.0	–
20	0.0	20.0	80.0	–	100.0	80.0	60.0	40.0	20.0	0.0	–
21	0.0	100.0	75.0	–	37.5	0.0	0.0	0.0	0.0	0.0	–
22	0.0	100.0	100.0	–	71.4	28.6	0.0	0.0	0.0	0.0	–
23	0.0	100.0	77.8	–	22.2	11.1	0.0	0.0	0.0	0.0	–
24	0.0	100.0	85.7	–	71.4	57.1	42.9	28.6	14.3	0.0	–
25	0.0	100.0	60.0	–	0.0	0.0	0.0	0.0	0.0	0.0	–
26	0.0	75.0	100.0	–	50.0	25.0	0.0	0.0	0.0	0.0	–
27	0.0	100.0	90.0	–	80.0	60.0	50.0	40.0	30.0	15.0	–
28	0.0	60.0	100.0	–	80.0	60.0	20.0	0.0	0.0	0.0	–
29	0.0	100.0	100.0	–	0.0	0.0	0.0	0.0	0.0	0.0	–
30	0.0	100.0	71.4	–	42.9	28.6	14.3	0.0	0.0	0.0	–
31	0.0	100.0	47.1	–	23.5	11.8	0.0	–	0.0	–	–
32	0.0	100.0	100.0	–	33.3	33.3	0.0	–	0.0	–	–
33	0.0	100.0	42.9	–	14.3	0.0	0.0	–	0.0	–	–
34	0.0	100.0	75.0	–	0.0	0.0	0.0	–	0.0	–	–
35	0.0	100.0	68.8	–	43.8	37.5	25.0	–	18.8	–	–
36	0.0	100.0	100.0	–	85.7	71.4	42.9	–	28.6	–	0.0
37	0.0	100.0	66.7	–	33.3	33.3	0.0	–	0.0	–	0.0
38	0.0	100.0	75.0	–	50.0	25.0	12.5	–	0.0	–	0.0
39	0.0	100.0	83.3	–	50.0	16.7	0.0	–	0.0	–	0.0
40	0.0	100.0	100.0	–	75.0	50.0	25.0	–	0.0	–	0.0
41	0.0	66.7	100.0	83.3	16.7	–	0.0	–	0.0	–	–
42	0.0	100.0	90.0	70.0	40.0	–	30.0	–	10.0	–	–
43^†^	0.0	0.0	0.0	0.0	0.0	–	0.0	–	0.0	–	–
44	0.0	100.0	0.0	0.0	0.0	–	0.0	–	0.0	–	–
45	0.0	100.0	100.0	50.0	0.0	–	0.0	–	0.0	–	–
46	0.0	100.0	60.0	60.0	40.0	–	0.0	–	0.0	–	–
47	0.0	100.0	60.0	30.0	10.0	–	10.0	–	0.0	–	–
48	0.0	100.0	80.0	40.0	20.0	–	0.0	–	0.0	–	–
49	0.0	83.3	100.0	100.0	50.0	–	16.7	–	0.0	–	–
50	0.0	85.7	100.0	42.9	28.6	–	0.0	–	0.0	–	–
51	0.0	100.0	80.0	60.0	40.0	–	20.0	–	0.0	–	–
52	0.0	83.3	100.0	–	–	–	–	–	66.7	66.7	–
53	0.0	100.0	75.0	–	–	–	–	–	25.0	0.0	–
54	0.0	100.0	66.7	–	–	–	–	–	16.7	0.0	–
55	0.0	100.0	83.3	–	–	–	–	–	0.0	0.0	–
56	0.0	100.0	80.0	–	–	–	–	–	0.0	0.0	–
57	0.0	20.0	100.0	–	–	–	–	–	20.0	0.0	–
58	0.0	75.0	100.0	–	–	–	–	–	50.0	0.0	–
59	0.0	100.0	75.0	–	–	–	–	–	37.5	0.0	–
60	0.0	100.0	75.0	–	–	–	–	–	37.5	12.5	–
61	0.0	100.0	70.0	–	–	–	–	–	20.0	0.0	–
62	0.0	100.0	100.0	–	75.0	62.5	–	–	0.0	0.0	–
63	0.0	100.0	77.8	–	55.6	44.4	–	–	22.2	11.1	–
64	0.0	100.0	87.5	–	62.5	37.5	–	–	37.5	0.0	–
65	0.0	100.0	87.5	–	62.5	50.0	–	–	12.5	0.0	–
66	0.0	–	100.0	87.5	87.5	–	–	–	37.5	0.0	–
67	0.0	–	100.0	75.0	50.0	–	–	–	25.0	0.0	–
68	0.0	–	100.0	70.0	40.0	–	–	–	0.0	0.0	–
69	0.0	–	100.0	75.0	62.5	–	–	–	12.5	0.0	–
70	0.0	–	100.0	80.0	70.0	–	–	–	0.0	0.0	–
71	0.0	–	100.0	40.0	0.0	–	–	–	0.0	0.0	–
72	0.0	–	100.0	87.5	87.5	–	–	–	50.0	0.0	–
73	0.0	–	100.0	88.9	77.8	–	–	–	33.3	0.0	–
74	0.0	–	100.0	62.5	50.0	–	–	–	25.0	12.5	–

*Dashes indicate subjects were not sampled at these time points.

^†^Subject failed to complete the self-assessment form.

### Physical assessment of impairment: horizontal gaze nystagmus

**Figure 2:**
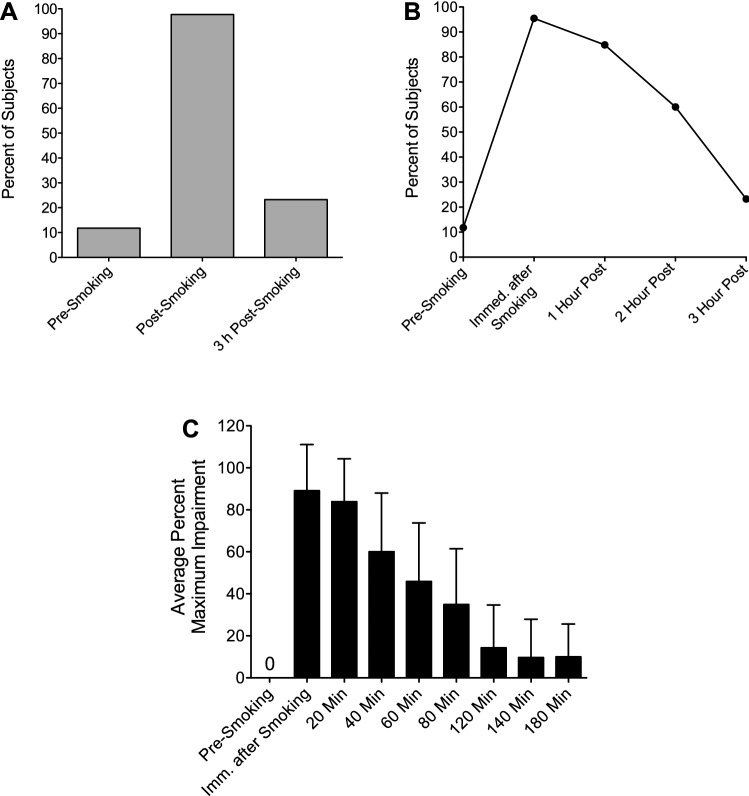
Pre- and post-smoking incidence of horizontal gaze nystagmus and correlation with impairment. (**A**) Overall incidence of nystagmus within the three-hour impairment window (*N* = 34 pre-smoking; *N* = 44 post-smoking; *N* = 43 3 h post-smoking); (**B**) incidence of nystagmus within the three-hour impairment window over time (*N* = 34 pre-smoking; *N* = 44 immediately after smoking; *N* = 33 1 h post-smoking; *N* = 10 2 h post-smoking; *N* = 43 3 h post-smoking); (**C**) average percent maximum impairment (+ SD) pre-smoking to three hours post-smoking (*N* = 74).

Horizontal gaze nystagmus (HGN) was assessed in 44 subjects. Nystagmus, both horizontal and vertical, refers to the involuntary jerking of the eyes as they gaze up or down. Someone experiencing nystagmus is unaware of its occurrence. Horizontal gaze nystagmus was evaluated prior to smoking cannabis and at various time points up to three hours post-smoking. The results showed that 43 of the 44 subjects (98%) exhibited HGN after smoking cannabis within the three-hour impairment window (Fig. 2A). Within the first 20 min after smoking, 42 of the 44 subjects (95.5%) exhibited HGN (Fig. 2B). These findings corresponded to the self-assessed impairment data, which showed that the average percent maximum impairment peaked within the first 20 min after smoking (Fig. 2C). At three hours post-smoking, the incidence of HGN had fallen to about 23% (10/43 subjects), compared to about 12% prior to smoking (Fig. 2B), which also corresponded well to the self-assessed impairment data showing that the average percent maximum impairment had fallen to approximately 10% three hours after smoking (Fig. 2C).

### Baseline detection of ∆^9^-THC and other cannabinoids in exhaled breath

Out of a total of 34 evaluable subjects, 23 (67.6%) had detectable ∆^9^-THC in their breath at baseline prior to smoking (Table 2), which is consistent with recent reports by Lynch et al.^10^ and Olla et al.^11^ showing 100% pre-smoking detection rates of ∆^9^-THC in breath in separate 20-subject and 23-subject studies, respectively. This finding provides further evidence that ∆^9^-THC cannot be solely relied upon as an indicator of recent cannabis use through inhalation within the impairment window. Other cannabinoids detected in exhaled breath prior to smoking included cannabinol (CBN) in one subject, cannabigerol (CBG) in two subjects, and cannabigerolic acid (CBGA) in four subjects (see Table 2). Notably, none of the 44 subjects were found to have detectable levels of any ∆^9^-THC metabolites in their breath either before or after smoking.

**Table 2:** Presence of key cannabinoids in exhaled breath before and after smoking.

Cannabinoid parameters	Percent (%) positivity		
	Baseline (pre-smoking)*	≤ 60 min after smoking	> 60 min after smoking^†^
∆^9^-THC	23/34 (67.6%)	40/40 (100%)	37/40 (92.5%)
CBN	1/34 (2.9%)	37/40 (92.5%)	4/40 (10.0%)
CBC	0/34 (0%)	39/40 (97.5%)	0/40 (0%)
CBG	2/34 (5.9%)	37/40 (92.5%)	1/40 (2.5%)
CBGA	4/34 (11.8%)	18/40 (45.0%)	4/40 (10.0%)
∆^9^-THCV	0/34 (0%)	36/40 (90.0%)	0/40 (0%)

*Pre-smoking samples were not collected from 10 subjects.

^†^No data beyond 60 min post-smoking in 4 subjects.

### Detection of key cannabinoids in breath

Table 2 shows the percent positivity of six cannabinoids prior to smoking, within the first hour post-smoking during peak impairment, and more than one hour post-smoking in subjects’ exhaled breath samples. In particular, CBN, cannabichromene (CBC), CBG, and ∆^9^-tetrahydrocannabivarin (∆^9^-THCV) all have a much greater incidence in breath during peak impairment compared to pre-smoking. Interestingly, CBC and ∆^9^-THCV were detected in breath only during the peak impairment window, making these two cannabinoids key indicators of recent cannabis use through inhalation. Stability of the analytes within the collection devices was previously established (see Supplementary Table S3). See Supplementary Tables S4 and S5 for summaries of intra-day and inter-day LC-HRMS method accuracy and precision, respectively, and Supplementary Table S6 for a summary of assay extraction efficiency (analyte recovery).

### Parameters of the recent use breath test

After analyzing the breath samples from all 44 subjects, 10 pharmacologic parameters were found to be associated with recent cannabis use based on the two-point breath sampling strategy, with additional parameters still being investigated. These included the presence of CBN, CBC, CBG, ∆^9^-THCV, and CBGA (summarized in Table 2), and short half-lives (< 60 min) for ∆^9^-THC, CBN, CBC, CBG, and ∆^9^-THCV (see Table 3). Compared to the average ∆^9^-THC half-life measured between 60 and 80 min post-smoking (outside of the one-hour peak impairment window post-smoking), the average ∆^9^-THC half-lives measured from immediately after smoking to 20 min post-smoking (*p* < 0.0001), and from 20 to 60 min post-smoking (*p* = 0.0201), were significantly shorter. Pre-smoking, half-lives were calculable for ∆^9^-THC in only three subjects. Raw half-life data for each cannabinoid at each time interval are shown in Supplementary Tables S7–S11. Table 3 and Supplementary Tables S7–S11 are based on the first 35 (#31-65) of the 44 subjects. The last nine subjects (#66-74) were sampled using a back-to-back sampling strategy and were treated separately see “Feasibility of Back-to-Back Exhaled Breath Sample Collection Strategy” below.

**Table 3:** Summary of cannabinoid half-lives in breath post-smoking.

Cannabinoid	Average half-life (minutes ± SD)						
	Minutes post-smoking						
	Pre-smoking	0–10	0–20	20–40	20–60	40–60	60–80
∆^9^-THC	28.8 ± 28.7 (*N* = 3)	4.4 ± 2.2 (*N* = 10)	4.2 ± 2.1* (*N* = 35)	8.1 ± 2.3 (*N* = 9)	12.8 ± 8.8^†^ (*N* = 23)	24.4 ± 25.4 (*N* = 9)	35.0 ± 41.7 (*N* = 6)
CBN	–^‡^	3.7 ± 2.1 (*N* = 11)	3.7 ± 3.0 (*N* = 20)	–	35.2 (*N* = 2)	12.8 (*N* = 1)	–
CBC	–	3.9 ± 2.5 (*N* = 10)	3.8 ± 2.3 (*N* = 20)	–	–	–	–
CBG	–	3.6 ± 2.1 (*N* = 11)	3.7 ± 3.1 (*N* = 20)	–	–	–	–
∆^9^-THCV	–	4.2 ± 3.9 (*N* = 9)	2.9 ± 0.7 (*N* = 6)	–	–	–	–

*p < 0.0001 compared to 60–80 min; ^†^*p* = 0.0201 compared to 60–80 min (two-tailed t-test with Bonferroni’s adjustment for multiple comparisons; α = 0.025).

^‡^Half-life not calculable.

### Breath-based test results

Within the first hour after smoking, which is the period of peak impairment, all 44 subjects (100%) were breath test positive, meaning they all exhibited a short ∆^9^-THC half-life (ranging from 1.0 to 19.1 min), and one or more other indicators of recent use in breath as described above under “Parameters of the Recent Use Breath Test.” Pre-smoking, all 34 subjects sampled were breath test negative, with two subjects exhibiting only short ∆^9^-THC half-lives. By itself, a short ∆^9^-THC half-life is insufficient evidence of recent use within the impairment window because ∆^9^-THC is commonly seen in the breath of non-recent cannabis users. Additional evidence is needed to confirm recent use.

### Breath and blood test

**Figure 3:**
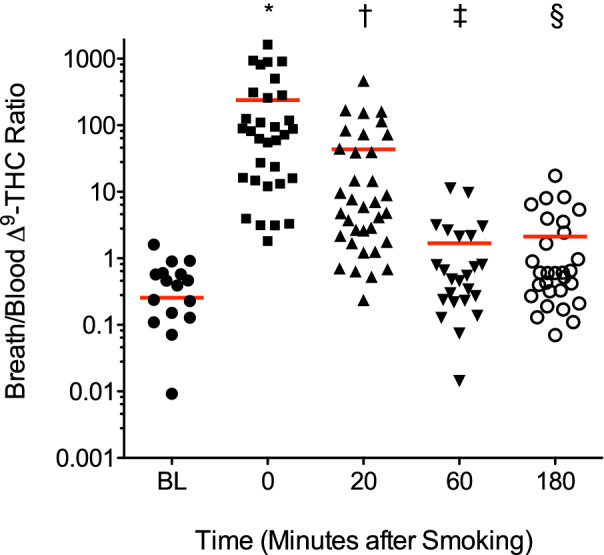
Breath/blood ∆^9^-THC ratios. Red bars indicate mean values. *N* = 29 (pre-smoking baseline; BL); *N* = 32 (immediately after smoking; 0 min); *N* = 35 (20 min post-smoking); *N* = 24 (60 min post-smoking); *N* = 31 (180 min post-smoking). Zero values (13 at BL and 2 at 180 min) could not be shown (logarithmic scale). Due to assay quantification limits, three values (two immediately after smoking and one at 60 min post-smoking) were excluded. *(*p* = 0.0015), ^†^(*p* = 0.0108), ^‡^(*p* = 0.0107), ^§^(*p* = 0.0091) compared to BL (two-tailed t-test with Bonferroni’s adjustment for multiple comparisons; α = 0.0125).

We hypothesized that the incorporation of both exhaled breath and blood into a comprehensive recent cannabis use test would confirm recent use of inhaled cannabis within the impairment window, improving the accuracy of the two-point breath-based testing method. For this purpose, blood samples were collected from the 44 subjects in addition to breath samples and analyzed for ∆^9^-THC and other cannabinoids. An eleventh recent use parameter that emerged was the breath/blood ratio of ∆^9^-THC, which is a key confirmatory indicator of recent use. This ratio was computed by dividing the ∆^9^-THC peak area ratio to the internal standard (IS) in breath by the corresponding peak area ratio in blood. We found that all of these ratios were < 2 when measured pre-smoking, while all values were > 2 when assessed immediately after smoking. Compared to the average pre-smoking ratio, the average breath/blood ∆^9^-THC ratios measured immediately after smoking (*p* = 0.0015), 20 min post-smoking (*p* = 0.0108), 60 min post-smoking (*p* = 0.0107), and 180 min post-smoking (*p* = 0.0091) remained significantly greater (see Fig. 3). In the two subjects who exhibited short ∆^9^-THC half-lives in breath pre-smoking, their breath/blood ∆^9^-THC ratios were 0.01 and 0.57, confirming that they had not recently used cannabis within the impairment window.

### Breath and blood test results

Within the first hour after smoking, which is the period of peak impairment, all 44 subjects (100%) were breath and blood test positive, meaning they all exhibited a breath/blood ∆^9^-THC ratio ≥ 2, in addition to a short ∆^9^-THC half-life and one or more other indicators of recent use in breath. Overall, using the two-point breath and one-point blood test, 0/34 subjects (0%) tested pre-smoking were positive, indicating no false positive results, and 44/44 subjects (100%) were positive inside the three-hour impairment window. This comprehensive test, which incorporates a breath/blood ∆^9^-THC ratio (Fig. 3) and the presence and half-lives of key cannabinoids in breath (Table 3), definitively established recent cannabis use within the impairment window.

### Correlation between impairment and recent use parameters in exhaled breath

All 44 subjects from whom breath samples were collected self-reported peak impairment within the first 20 min after smoking (Table 1), which coincided with the shortest cannabinoid half-lives (Table 3) and peak incidence of horizontal gaze nystagmus (Fig. 2A,B).

### Feasibility of back-to-back exhaled breath sample collection strategy

Evidence of recent cannabis use by inhalation dissipates rapidly in breath, and thus allowing 20 min between samplings, which was employed in most of the subjects in this study, may result in loss of valuable data. In nine of the 44 subjects (#66-74) from whom breath samples were collected, a back-to-back sample collection strategy was employed whereby two breath samples were collected in quick succession at 20 and 40 min post-smoking. Approximately 2 min elapsed between the collection of each sample. The very short half-lives observed for ∆^9^-THC, CBN, and other cannabinoids, approximately 6 min or less (see Supplementary Table S12), in exhaled breath would allow for rapid sampling. The pharmacokinetic data from these nine subjects showed that this strategy is feasible. This approach could save a great deal of time and potentially result in more effective testing.

## Discussion

As legalization of medicinal and recreational cannabis continues to expand throughout the U.S. and worldwide, so too does the need for an objective means of determining recent cannabis use and impairment, which cannot be established using currently available breath-based or blood-based testing methods. To address this critical unmet need for the benefit of law enforcement, employers, and others for whom determining recent cannabis use and impairment is a necessity, the goal of our study was to devise a comprehensive test based on pharmacological changes in ∆^9^-THC and other cannabinoids that occur with time in exhaled breath and blood, a test that improves the accuracy of breath-based testing by incorporating a confirmatory blood test to prevent false positive results. It was hypothesized that a two-sample testing strategy (collecting two samples separated by a known time interval) could be used to detect cannabinoids in breath during their distribution phases, which occur only during the first few hours after smoking. A confirmatory blood test could be used to compare relative levels of cannabinoids in breath. Higher relative levels of cannabinoids in breath compared to blood would be indicative of very recent use because it is not possible for higher relative levels of cannabinoids to redistribute back into the breath from the blood. Coupled with physical and subject self-assessments of impairment, it was believed that the results of the test could also be tied to recent use within the impairment window. As will be discussed later, an alternate approach is needed for oral cannabis use as opposed to inhalation due to differences in cannabinoid pharmacokinetics.

Testing methodologies currently being employed for cannabis use include urine, blood, and oral fluid (saliva) analysis. Urine testing is an excellent means of determining prior use, and it has traditionally been employed, for example, in routine workplace drug screening and drug use monitoring in parolees. However, in frequent cannabis users, metabolites of ∆^9^-THC such as 11-nor-9-carboxy-∆^9^-THC may be detectable in urine for several weeks after last use, and ∆^9^-THC itself has been detected in urine up to 24 days following most recent use^15,16^. While different approaches to utilizing urinary cannabinoid data have been studied in regard to determining recent use^15^, none of these methods is capable of determining cannabis use within the impairment window, and thus urine testing is not useful in this context.

In cases of suspected cannabis DUI, blood testing has been the gold standard. There are, however, a number of reasons why blood testing, at least as it is currently being employed, is inadequate for establishing recent cannabis use within the impairment window. In law enforcement settings, blood samples are typically not collected until approximately 1.5 to 4 h following a traffic stop or collision^17,18^, a time when plasma ∆^9^-THC concentrations may have already fallen to very low or undetectable levels. Furthermore, current testing approaches utilize one blood draw, which will at best provide a single measure of ∆^9^-THC and metabolite concentrations. This is problematic because recent research has shown that there is no meaningful correlation between impairment and ∆^9^-THC blood levels^19–21^. This has become a real issue in U.S. states and other countries that have arbitrarily established legal “per se” ∆^9^-THC limits for the determination of cannabis impairment. It has also been shown that ∆^9^-THC can be detected in the blood of chronic users after 30 days of abstinence^22^, and that exercise can increase the blood level of ∆^9^-THC due to release from fat stores^23^.

Compared to urine and blood testing, oral fluid offers a non-invasive means of assessing drug use. A number of different drugs of abuse and their metabolites can be detected in oral fluid, including ∆^9^-THC^24^. Oral fluid testing devices are already being employed for DUI testing in countries such as Canada, Australia, Norway, and the U.S. Similar to blood, levels of ∆^9^-THC in oral fluid can persist well beyond the window of impairment^25–27^. In frequent cannabis users, ∆^9^-THC was detected in oral fluid for an average of 61 h after smoking^24^. The oral cavity is also subject to contamination from environmental smoke^28,29^, which could result in a false positive test result. In field testing of oral fluid performed by a popular commercial testing device, false positive and false negative rates of 14.5% and 13.4%, respectively, were encountered for cannabis use^30^. A similar rate of false positive test results was reported for another competing oral fluid testing device in a clinical study evaluating oral fluid cannabinoid pharmacokinetics following consumption of cannabis edibles^31^. While oral fluid screening may be useful in a workplace setting or for routine drug use monitoring in parolees, for example, it cannot be used to reliably assess recent use within the impairment window.

In the present study, all 44 subjects (100%) from whom both blood and breath samples were collected tested positive for recent use within the first hour after smoking, which is the time of peak impairment, using the two-point breath and one-point blood test. Pre-smoking, 0% (0/34) of subjects tested positive for recent use, indicating no false positive test results. A positive test result using the two-point breath and one-point blood test indicates that a subject has used cannabis recently through inhalation, i.e., smoking or vaping, and that they are within the three-hour impairment window. Interestingly, approximately 68% of these subjects had detectable levels of ∆^9^-THC in their breath at baseline prior to smoking, in agreement with recent reports by Lynch et al.^10^ and Olla et al.^11^. This finding suggests that the mere presence of ∆^9^-THC in breath does not conclusively demonstrate recent use within the impairment window, which could prove to be a major shortcoming of the commercial cannabis breathalyzers currently in development.

A potential limitation of the recent use breath test is the possibility for false negative test results. As already pointed out, evidence of recent cannabis use in breath declines rapidly beyond the first hour after smoking, so it is possible that breath samples collected during the second or third hour after cannabis inhalation may no longer exhibit sufficient evidence of recent use, even though the subject may still be impaired. A false negative result obviously favors the test subject, while a false positive result could have potentially serious consequences, including wrongful termination of employment and prosecution. With this test, it is possible to observe short ∆^9^-THC half-lives in breath and breath/blood ∆^9^-THC ratios ≥ 2 when concentrations of ∆^9^-THC are very low, at or near assay limits of detection. In order to avoid false positive test results due to assay variation at very low levels, it is important to ensure that concentrations fall within the validated range of the assay. It may be practical to set a 1.0-ng/mL ∆^9^-THC cutoff level to avoid false positives due to concentrations near detection limits.

In our study, a wide variation was observed in pre-smoking ∆^9^-THC blood concentrations, which ranged from undetectable to as high as 80 ng/mL, in the absence of impairment, which may be attributable to tolerance and variable usage patterns. After smoking to the desired effect, however, all subjects became impaired, which was correlated with nystagmus. The described two-point breath test was able to accurately detect recent use within the impairment window in subjects with a background of frequent cannabis use. Because of interindividual variation in tolerance, frequency of use, and metabolism among cannabis users, it is possible to observe impairment beyond 3 h post-smoking.

As we observed in our study, evidence of recent cannabis use in breath dissipates rapidly. It is thus critical that breath samples are collected as soon as possible following a workplace incident, for example, or once a need for testing arises, in order to prevent the loss of breath evidence. On-site breath collection using a device such as the one employed in this study can easily be performed without specialized training. Likewise, the blood sample can be collected on site using one of the commercially available capillary blood draw devices designed to be used without specialized training. For detecting recent cannabis use by inhalation, a two-point breath test is the most practical application. In the event that a test subject is positive for only a short ∆^9^-THC half-life in breath, the matching blood sample can optionally be used to confirm recent use if desired. A matching blood sample would always be collected to allow testing for other drugs that can induce impairment, including prescription drugs, and it can be additionally analyzed for cannabinoid content if needed.

While the focus of this test has been on detection of recent inhaled cannabis use, it should be emphasized that the two-point breath and one-point blood test is not limited to just cannabis. The same testing strategy we employed for cannabis in the present study may also prove to be useful for detecting recent use of other impairing drugs such as methamphetamine, phencyclidine, and cocaine that can be administered through vaporization. Exhaled breath testing has already been proven useful for detecting multiple drug types^32,33^, and this test allows the simultaneous testing for cannabis as well as other potentially impairing drugs in both breath and blood. Potential applications include sports medicine, enforcement of workplace drug policy, and law enforcement.

Another limitation of the test as currently designed is that it can detect recent use of cannabis only through inhalation within the impairment window. Further study is needed to detect recent use and impairment following oral consumption of cannabis products. Hypothetically, a similar strategy utilizing two blood samples could be deployed for detecting recent use of orally administered cannabis as well as other orally administered impairing drugs. It is well known that cannabinoid pharmacokinetics differ depending on the route of administration. Because ∆^9^-THC metabolites cannot be detected in breath, the blood may contain critical information pertaining to recent use, including concentrations of glucuronide metabolites and changes in the ratios of the ∆^9^-THC metabolites such as 11-hydroxy-∆^9^-THC and 11-nor-9-carboxy-∆^9^-THC to ∆^9^-THC and to each other, as previously reported^14,34^.

In conclusion, a new test for recent cannabis use and impairment, based on two-point breath sampling with or without a one-point confirmatory blood test, has been developed that can accurately detect whether a subject has used cannabis through inhalation within the three-hour impairment window, regardless of the potency of the cannabis chemovar smoked, with no false positive results. The test is based on multiple parameters, including cannabinoid half-lives, which confirm distribution phase kinetics, the presence of key cannabinoids that are observed only after smoking, and a blood test to determine the breath/blood ∆^9^-THC ratio, which confirms whether the test subject was within the impairment window post-smoking. It is our belief, based on our research, that this test provides an answer to the unmet need of determining recent cannabis use, which in turn protects the public from a safety standpoint by detecting inappropriate use, e.g., DUI, while at the same time protecting responsible cannabis users such as medicinal users from wrongful termination and prosecution. This test may finally help bring an end to cannabis discrimination.

## Materials and methods

### Clinical study

A total of 74 subjects were recruited to perform a study designed to develop a comprehensive test that confirms recent use of inhaled cannabis within the impairment window. All subjects received financial compensation for their participation. This study was performed under a clinical protocol approved by the Cancer Immunotherapy Research Institute IRB (assurance #FWA00029851), and all research activities were conducted in accordance with the Declaration of Helsinki. Written informed consent was obtained from all subjects prior to their participation, and a copy of the signed informed consent form was provided to each subject.

### Inclusion criteria

To be included, a subject must have been a male or female cannabis user at least 21 years of age. Prior to their scheduled participation, they must have used within the previous 24 h, but not within the last 12 h. Upon entry, subjects were asked to complete a questionnaire requesting their age, sex, race, height, weight, cannabis use history (time since last use, number of days used in the last 14 days, how often they use cannabis, number of years of cannabis use), their primary route of cannabis use, whether or not they use tobacco and alcohol, and any medications or supplements they are taking.

### Cannabis administration

Each subject was given a single cannabis cigarette and instructed to smoke as much of it as possible within a 10-min period. Cigarettes containing 500 mg of dried cannabis flower with a ∆^9^-THC content ranging from 8.5 to 28.4% were prepared immediately before each smoking session. Cannabis supplies were legally obtained from licensed retail establishments in the Sacramento, CA region. A wide variety of chemovars was included to account for the variability in potencies available in numerous cannabis retail establishments in the various U.S. states where recreational and/or medicinal cannabis has been legalized.

### Blood draw schedule

Blood samples were obtained from all 74 subjects. To establish baseline cannabinoid levels prior to smoking, two capillary blood samples were collected 20 min apart. Post-smoking blood samples were collected immediately after smoking and then at 20, 40, 60, 80, 100, 120, 140, 160, 180, and 200 min post-smoking. Capillary blood (50–100 µL) was collected into BD Microtainer tubes containing lithium heparin anticoagulant (Thermo Fisher Scientific; Waltham, MA) after pricking subjects’ fingers using 17-gauge lancets (McKesson Medical-Surgical Inc., Richmond, VA). Some capillary blood samples were drawn using automated collection devices from Tasso, Inc. (Seattle, WA) and Seventh Sense Biosystems, Inc. (Medford, MA) equipped with sample reservoirs containing lithium heparin. These devices are designed to draw approximately 100–150 µL of whole blood over a period of 1–3 min.

### Breath collection schedule

Breath samples were obtained from a total of 44 subjects (#31-74). The other 30 subjects (#1-30) had only blood samples collected because the original design was to develop a blood-based cannabis recent use test. Data from these first 30 subjects showed that while a blood-based test is very good at detecting recent use, there was also an unacceptable rate of false positives outside of the impairment window. At that point, it was decided that an additional component, exhaled breath, was needed to more accurately detect recent cannabis use within the impairment window. Self-assessed impairment information (see below) was still collected from these 30 subjects.

To establish baseline cannabinoid levels, two breath samples were collected 20 min apart prior to smoking. Post-smoking breath samples were collected immediately after smoking, and then at 10, 20, 30, 40, 50, 60, 80, 120, 180, and 240 min post-smoking in the first 35 subjects (#31-65). In the last nine subjects (#66-74), back-to-back breath samples were collected at 20 and 40 min post-smoking to evaluate the feasibility of this sampling strategy. Breath sample collection devices were provided by Sensabues AB (Stockholm, Sweden). These self-contained, single-use devices contain an electrostatic polymer filter and are designed to collect about 20 L of exhaled breath through normal breathing. During sample collection, subjects were seated and instructed to blow through the device until the attached bag was fully inflated. The time required for sample collection was approximately 2–3 min. No instances of hyperventilation or other breathing abnormalities were observed. Devices were kept sealed in their original packaging until immediately before use to prevent contamination and used according to the manufacturer’s instructions. The smoking room was well ventilated and allowed to clear for at least 24 h prior to each subject smoking session. Immediately after sample collection, the devices were resealed, removed from the collection area, and held at room temperature (20–25 °C). All samples were extracted and analyzed within 24 h of collection.

### Self-assessment of impairment

All 74 subjects, including the first 30 subjects from whom only blood samples were collected and the 44 subjects from whom both breath and blood samples were collected, were asked to self-assess their level of impairment before smoking and at each designated time point after smoking based on a scale ranging from 0 (not impaired) to 10 (very impaired). To normalize, impairment data were expressed as a percentage relative to each individual subject’s maximum reported impairment level.

### Physical assessment of impairment: horizontal gaze nystagmus

In this study, a subset of 44 subjects were evaluated for horizontal gaze nystagmus (HGN) as a physical indicator of impairment. Horizontal gaze nystagmus refers to the involuntary movement or jerking of the eyes as they gaze to either side, and it is a component of standardized field sobriety testing^35^. In this particular test, subjects are asked to keep their head still and follow a slowly moving horizontal object positioned in front of their face using their eyes only. Both eyes are observed for lack of smooth pursuit, nystagmus at maximum eye deviation (45°), and the onset of nystagmus prior to a 45° deviation. The presence or absence of resting nystagmus is also noted.

### Analytical methods

#### Chemicals and reagents

Eight of the 10 cannabinoid analytes [∆^9^-THC, CBN, cannabigerol (CBG), cannabigerolic acid (CBGA), ∆^9^-tetrahydrocannabinolic acid A (∆^9^-THCA), ∆^9^-tetrahydrocannabivarin (∆^9^-THCV), 11-OH-∆^9^-THC, and 11-nor-9-carboxy-∆^9^-THC] and the internal standard (IS; ∆^9^-THC-D_3_) were obtained as certified reference materials (CRMs) manufactured by Cerilliant (Round Rock, TX). The 8β,11-diOH-Δ^9^-THC metabolite was obtained from ElSohly Laboratories, Inc. (Oxford, MS). Cannabichromene (CBC) was obtained as a CRM from Cayman Chemical (Ann Arbor, MI). When not in use, concentrated stock solutions of these agents and working solutions made therefrom were stored at – 20 °C.

Acetonitrile, formic acid, methanol, and *n*-hexane were purchased from Thermo Fisher Scientific and were of LC/MS grade. Ethyl acetate (Acros Organics) was purchased from Thermo Fisher Scientific and was of spectroscopy grade (> 99.5%). High purity water (18.2 MΩ) required for preparing the mobile phase and for sample extraction was produced using an EMD Millipore Simplicity water purification system. When not in use, these agents were stored at room temperature (20–25 °C). Nitrogen (N_2_), supplied as a cryogenic liquid in a 230L dewar at a purity of 99.998%, or as compressed nitrogen gas at a purity of 99.999% in T-type cylinders, was obtained from Praxair (Danbury, CT).

#### Analysis of cannabinoids in exhaled breath

A previously validated LC-HRMS analytical method for the quantification of the cannabinoids ∆^9^-THC, CBN, CBC, and ∆^9^-THCV in exhaled breath was used for the analysis of study samples. Additional cannabinoids analyzed included ∆^9^-THCA, CBG, and CBGA. For the preparation of calibration standards, sufficient quantities of the matrix (breath collection devices) were obtained from SensAbues AB. Breath collection devices were kept at room temperature (20–25 °C) within their original packaging to prevent contamination.

Concentrated standard calibration solutions were prepared in methanol at 37.5, 75, 150, 375, 750, and 1500 ng/mL of all cannabinoids combined. Following extraction and reconstitution, final standard concentrations were 2.5, 5.0, 10, 25, 50 and 100 ng/mL. The IS solution was prepared in methanol at a concentration of 75 ng/mL. To prepare calibration standards for extraction, 5 µL of the IS working solution and 5 µL of the appropriate calibration standard solution were added directly onto the corresponding filter pad inside the breath collection device. After extraction, the final concentration of the IS was 5 ng/mL (75 µL final volume). Study samples were prepared by spiking with 5 µL IS solution.

To extract cannabinoids from the breath collection devices, 2 mL of methanol were aliquoted through each device and filter housing. After adding methanol, approximately 5 min were allowed for all of the solvent to pass through the filter pads. Next, two 2.5-mL aliquots of methanol were passed through the breath devices. Using a 60-mL syringe, approximately 120 cm^3^ of air was pushed through each device to force all residual methanol through the filter pads. The sample breath collection devices were then removed and the glass tubes were placed in an N-Evap Model 112 analytical nitrogen evaporator (Organomation Associates, Berlin, MA). The eluate was evaporated to dryness under a gentle stream of nitrogen gas, with the water bath temperature set to approximately 50 °C. Once evaporation was complete, the samples were allowed to cool to room temperature and reconstituted by adding 75 µL of a solution containing 75% acetonitrile and 25% water with 0.1% formic acid. The samples were then transferred to a glass microinsert-equipped autosampler vial and placed in the autosampler compartment for analysis according to the method. TraceFinder software performed all required analyses. The chromatographic conditions for the analysis of cannabinoids in exhaled breath were the same as previously described^14^. To calculate half-life, the standard equation t_1__/2_ = 0.693/k was used, where k = ln (start value/end value)/t, and where t = time in minutes. The starting and ending values were determined by calculating the peak area ratios of analyte to internal standard.

#### Analysis of cannabinoids in blood

Extraction and analysis of ∆^9^-THC, its major metabolites, and other cannabinoids in whole blood was performed according to a validated method as previously described^14^. Briefly, 50 µL of each sample was mixed with 100 µL of high-purity water in a 1.5-mL microcentrifuge tube and spiked with 5.0 µL of IS solution. To extract, 500 µL of a solution containing 90% *n*-hexane and 10% ethyl acetate (v/v) was added to each sample, followed by vortexing for 30 s. Samples were then centrifuged at 9300 rcf for 10 min. The supernatant was transferred to a 16 mm × 125 mm borosilicate glass tube and evaporated to dryness under a gentle stream of nitrogen gas at 50 °C. Samples were then reconstituted in 75 µL of a solution composed of 65% acetonitrile, 35% water, and 0.1% formic acid and analyzed by LC-HRMS. Supplies of whole blood needed to prepare calibration standards were obtained from a reliable, cannabis-free donor and kept refrigerated (2–8 °C) for up to six weeks.

The LC-HRMS system consisted of a Thermo Scientific Vanquish ultra-high-performance liquid chromatography (UHPLC) system and a Thermo Scientific Q Exactive mass spectrometer. All analytical data were collected and processed using TraceFinder version 4.1 software (Thermo Fisher Scientific). The mass spectrometer and UHPLC system were configured as previously described^14^.

### Statistical methods

Differences between average breath/blood ∆^9^-THC ratios and cannabinoid half-lives were evaluated using a two-sample equal variance Student’s t-test with a two-tailed distribution and significance level of 0.05. Bonferroni’s adjustment was employed to lessen the likelihood of false positive results, with the significance level being adjusted to α = 0.0125 when comparing the breath/blood ∆^9^-THC ratios (four tests) and α = 0.0250 when comparing average cannabinoid half-lives (two tests).

## Supplementary Information

Supplementary Information.

## Data Availability

All datasets generated and/or analyzed during the present study are either available in the main text and supplementary materials, or can be obtained from the corresponding author on reasonable request.
