# Supplementary material for: A comprehensive breath test that confirms recent use of inhaled cannabis within the impairment window
Source: Sci Rep. 2021 Nov 23;11:22776. doi: 10.1038/s41598-021-02137-x (PMC8611040; doi:10.1038/s41598-021-02137-x)
Supplement: Supplementary file 1 — Supplementary Information. [file 41598_2021_2137_MOESM1_ESM.pdf]

SUPPLEMENTARY MATERIALS

A Comprehensive Breath Test that Confirms Recent Use of Inhaled Cannabis within the Impairment Window

Michael W. DeGregorio, Gregory T. Wurz, Edward Montoya, Chiao-Jung Kao

Supplementary Table S1. Clinical subject demographics

Subject	Age	Sex
1	23	M
2	22	M
3	42	M
4	23	M
5	21	M
6	24	M
7	23	F
8	29	M
9	23	M
10	23	M
11	23	F
12	25	M
13	23	F
14	23	M
15	24	M
16	29	M
17	25	M
18	23	M
19	23	M
20	42	M
21	23	M
22	24	M
23	24	M
24	23	F
25	21	M
26	23	M
27	25	M
28	24	M
29	23	M
30	29	F
31	23	M
32	22	M
33	29	F
34	23	M
35	23	M
36	22	F
37	29	M
38	24	M
39	22	F
40	42	M

Subject	Age	Sex
41	22	F
42	23	M
43	23	M
44	25	M
45	29	M
46	24	M
47	34	F
48	31	F
49	23	M
50	23	M
51	25	M
52	23	M
53	22	M
54	22	F
55	24	M
56	22	F
57	23	F
58	33	F
59	22	M
60	23	M
61	26	M
62	24	M
63	23	M
64	24	M
65	23	M
66	22	F
67	22	M
68	26	M
69	22	M
70	24	M
71	30	F
72	33	F
73	22	M
74	24	M

Supplementary Table S2. Self-assessed impairment raw data

	Self-Assessed Impairment Level (0-10) Post-Smoking (minutes)										
**Subject**	**Pre-Smoking**	**0**	**20**	**40**	**60**	**80**	**120**	**140**	**180**	**200**	**240**
**1**	0	4	6	—*	5	3	1	0	0	0	—
**2**	0	1	3	—	2	2	0	0	0	0	—
**3**	0	4	5	—	6	6	5	4	2	0	—
**4**	0	6.5	5.5	—	3	1.5	0.5	0.5	0	0	—
**5**	0	3	5	—	3	1	0	0	0	0	—
**6**	0	8	5	—	1	0	0	0	0	0	—
**7**	0	6	5	—	4	4	3	2	1	0	—
**8**	0	2	3	—	2	2	1	0	0	0	—
**9**	0	5	4	—	2	0	0	0	0	0	—
**10**	0	8	5	—	2	1	0	0	0	0	—
**11**	0	6	6	—	4	3	2	0	0	0	—
**12**	0	10	9	—	8	8	6	5	3	2	—
**13**	0	5	4	—	0	0	0	0	0	0	—
**14**	0	6	5	—	1	1	1	0	0	0	—
**15**	0	6	5	—	3	2	0	0	0	0	—
**16**	0	3	2	—	1	1	0	0	0	0	—
**17**	0	3	4	—	1	0	0	0	0	0	—
**18**	0	7	8	—	4	4	3	1	1	0	—
**19**	0	6	9	—	5	4	2	1	0	0	—
**20**	0	1	4	—	5	4	3	2	1	0	—
**21**	0	8	6	—	3	0	0	0	0	0	—
**22**	0	7	7	—	5	2	0	0	0	0	—
**23**	0	9	7	—	2	1	0	0	0	0	—
**24**	0	7	6	—	5	4	3	2	1	0	—
**25**	0	5	3	—	0	0	0	0	0	0	—
**26**	0	3	4	—	2	1	0	0	0	0	—
**27**	0	10	9	—	8	6	5	4	3	1.5	—
**28**	0	3	5	—	4	3	1	0	0	0	—
**29**	0	3	3	—	0	0	0	0	0	0	—
**30**	0	7	5	—	3	2	1	0	0	0	—
**31**	0	8.5	4	—	2	1	0	—	0	—	—
**32**	0	3	3	—	1	1	0	—	0	—	—
**33**	0	7	3	—	1	0	0	—	0	—	—
**34**	0	4	3	—	0	0	0	—	0	—	—
**35**	0	8	5.5	—	3.5	3	2	—	1.5	—	—
**36**	0	7	7	—	6	5	3	—	2	—	0
**37**	0	3	2	—	1	1	0	—	0	—	0
**38**	0	8	6	—	4	2	1	—	0	—	0

	Self-Assessed Impairment Level (0-10) Post-Smoking (minutes)									
**Subject**	**Pre-Smoking**	**0**	**20**	**40**	**60**	**80**	**120**	**140**	**180**	**200**	**240**
**39**	0	6	5	—	3	1	0	—	0	—	0
**40**	0	4	4	—	3	2	1	—	0	—	0
**41**	0	4	6	5	1	—	0	—	0	—	—
**42**	0	10	9	7	4	—	3	—	1	—	—
**43†**	0	0	0	0	0	—	0	—	0	—	—
**44**	0	1	0	0	0	—	0	—	0	—	—
**45**	0	2	2	1	0	—	0	—	0	—	—
**46**	0	5	3	3	2	—	0	—	0	—	—
**47**	0	10	6	3	1	—	1	—	0	—	—
**48**	0	5	4	2	1	—	0	—	0	—	—
**49**	0	5	6	6	3	—	1	—	0	—	—
**50**	0	6	7	3	2	—	0	—	0	—	—
**51**	0	10	8	6	4	—	2	—	0	—	—
**52**	0	5	6	—	—	—	—	—	4	4	—
**53**	0	8	6	—	—	—	—	—	2	0	—
**54**	0	6	4	—	—	—	—	—	1	0	—
**55**	0	6	5	—	—	—	—	—	0	0	—
**56**	0	5	4	—	—	—	—	—	0	0	—
**57**	0	1	5	—	—	—	—	—	1	0	—
**58**	0	6	8	—	—	—	—	—	4	0	—
**59**	0	8	6	—	—	—	—	—	3	0	—
**60**	0	8	6	—	—	—	—	—	3	1	—
**61**	0	10	7	—	—	—	—	—	2	0	—
**62**	0	8	8	—	6	5	—	—	0	0	—
**63**	0	9	7	—	5	4	—	—	2	1	—
**64**	0	8	7	—	5	3	—	—	3	0	—
**65**	0	8	7	—	5	4	—	—	1	0	—
**66**	0	—	8	7	7	—	—	—	3	0	—
**67**	0	—	8	6	4	—	—	—	2	0	—
**68**	0	—	10	7	4	—	—	—	0	0	—
**69**	0	—	8	6	5	—	—	—	1	0	—
**70**	0	—	10	8	7	—	—	—	0	0	—
**71**	0	—	5	2	0	—	—	—	0	0	—
**72**	0	—	8	7	7	—	—	—	4	0	—
**73**	0	—	9	8	7	—	—	—	3	0	—
**74**	0	—	8	5	4	—	—	—	2	1	—

*Dashes indicate subjects were not sampled at these time points
†Subject failed to complete the self-assessment form

Supplementary Table S3. Stability of target analytes in the breath collection device

	Analyte Stability (%) at Room Temperature (20-25°C)				
**Analyte**	**Day 3**	**Day 7**	**Day 10**	**Day 14**	**Day 30**
Δ^9-THC	91.3	81.2	82.9	66.2	55.6
CBN	104.5	100.0	107.3	87.9	74.4
CBC	100.6	89.1	89.9	82.2	75.6
CBG	97.5	87.6	97.8	75.0	56.5
Δ^9-THCV	79.7	68.1	69.8	56.5	47.1
CBGA	99.4	110.9	110.4	No data	114.3

Supplementary Table S4. Determination of cannabinoids in exhaled breath: intra-day method validation data

Concentration (ng/mL)	Parameter	Δ^9-THC	CBN	CBC	Δ^9-THCV
2.5	Accuracy (%)	-5.1	5.6	-0.2	4.7
(*N*=5)	Precision (%)	3.8	9.1	4.6	4.8
5.0	Accuracy (%)	-12.2	-9.6	-8.7	-10.4
(*N*=5)	Precision (%)	6.0	12.8	5.3	7.0
10	Accuracy (%)	-5.0	-11.7	-6.8	-7.1
(*N*=5)	Precision (%)	3.3	6.5	4.2	5.1
25	Accuracy (%)	-10.7	-13.3	-8.9	-9.3
(*N*=5)	Precision (%)	4.6	4.3	6.9	5.2
50	Accuracy (%)	2.0	0.9	1.4	1.8
(*N*=5)	Precision (%)	3.8	1.6	2.7	2.1
100	Accuracy (%)	-5.0	-7.4	-5.4	-5.2
(*N*=5)	Precision (%)	1.4	3.4	2.6	2.7

Supplementary Table S5. Determination of cannabinoids in exhaled breath: inter-day method validation data determined on 5 consecutive days

Concentration (ng/mL)	Parameter	Δ^9-THC	CBN	CBC	Δ^9-THCV
3.0	Accuracy (%)	-1.9	4.9	-11.9	-2.1
(*N*=5)	Precision (%)	12.6	24.8	18.2	10.0
45	Accuracy (%)	-0.7	-0.8	-5.0	-0.5
(*N*=5)	Precision (%)	8.0	11.7	5.2	11.4
90	Accuracy (%)	6.4	7.5	3.9	14.7
(*N*=5)	Precision (%)	5.3	7.3	12.6	11.4

Supplementary Table S6. Determination of cannabinoids in exhaled breath: extraction efficiency

Concentration (ng/mL)	Extraction Efficiency (%)			
	Δ^9-THC	CBN	CBC	Δ^9-THCV
20 (*N*=6)	63.8	56.7	70.2	54.2
80 (*N*=6)	51.4	42.7	52.0	45.0

Supplementary Table S7. Pre- and post-smoking Δ^9-THC half-lives in breath

	Δ^9-THC Half-Life (minutes)						
	Time Intervals Post-Smoking (minutes)						
**Subject**	**Pre-Smoking**	**0-10**	**0-20**	**20-40**	**20-60**	**40-60**	**60-80**
31	61.44	—*	4.73	—	19.19	—	—
32	—	—	3.26	—	7.48	—	—
33	—	—	6.23	—	8.79	—	—
34	—	—	4.38	—	16.31	—	—
35	17.39	—	5.55	—	9.71	—	21.22
36	7.67	—	3.13	—	9.00	—	24.58
37	—	—	4.36	—	4.00	—	—
38	—	—	3.57	—	—	—	—
39	—	—	3.96	—	6.11	—	—
40	—	—	3.28	—	12.58	—	—
41	—	5.40	4.81	6.86	10.34	20.96	—
42	—	9.40	5.79	9.50	10.21	11.03	—
43	—	5.50	6.25	8.39	10.72	14.83	—
44	—	2.92	2.83	8.20	28.63	—	—
45	—	3.32	3.15	—	—	—	—
46	—	4.50	3.24	—	46.20	17.17	—
47	—	—	7.15	6.28	7.82	10.37	—
48	—	5.19	3.39	6.44	9.12	15.58	—
49	—	2.23	2.02	7.03	10.68	22.22	—
50	—	1.75	2.52	6.34	11.86	91.24	—
51	—	3.52	3.45	13.55	14.64	15.93	—
52	—	—	2.39	—	—	—	—
53	—	—	2.89	—	—	—	—
54	—	—	3.32	—	—	—	—
55	—	—	2.46	—	—	—	—
56	—	—	3.56	—	—	—	—
57	—	—	6.03	—	—	—	—
58	—	—	3.39	—	—	—	—
59	—	—	4.35	—	—	—	—
60	—	—	3.89	—	—	—	—
61	—	—	3.29	—	—	—	—
62	—	—	1.65	—	10.29	—	6.81
63	—	—	13.90	—	7.49	—	24.18
64	—	—	4.30	—	11.05	—	119.06
65	—	—	4.66	—	11.22	—	14.18

*Dash indicates half-life not calculable

Supplementary Table S8. Post-smoking CBN half-lives in breath

	CBN Half-Life (min)			
	Time Intervals Post-Smoking (min)			
**Subject**	**0-10**	**0-20**	**20-60**	**40-60**
31	—*	—	—	—
32	—	2.70	—	—
33	—	—	—	—
34	—	—	—	—
35	—	4.99	8.81	12.76
36	—	2.67	—	—
37	—	—	—	—
38	—	—	—	—
39	—	—	—	—
40	—	—	—	—
41	5.01	—	—	—
42	8.77	5.43	—	—
43	5.29	—	—	—
44	2.24	2.74	61.67	—
45	2.69	3.43	—	—
46	3.86	—	—	—
47	2.21	2.66	—	—
48	4.28	3.08	—	—
49	1.76	1.55	—	—
50	1.50	2.38	—	—
51	2.99	—	—	—
52	—	—	—	—
53	—	—	—	—
54	—	2.79	—	—
55	—	1.97	—	—
56	—	2.94	—	—
57	—	—	—	—
58	—	2.82	—	—
59	—	3.60	—	—
60	—	3.31	—	—
61	—	2.82	—	—
62	—	1.46	—	—
63	—	15.60	—	—
64	—	—	—	—
65	—	4.12	—	—

*Dash indicates half-life not calculable

Supplementary Table S9. Post-smoking CBC half-lives in breath

	Half-Life (min)	
	Time Intervals Post-Smoking (min)	
Subject	0-10	0-20
31	—*	5.85
32	—	2.84
33	—	6.20
34	—	—
35	—	4.67
36	—	2.71
37	—	—
38	—	—
39	—	—
40	—	—
41	5.28	—
42	10.07	6.25
43	—	—
44	2.30	—
45	3.16	—
46	4.36	—
47	2.20	2.73
48	4.54	3.16
49	1.80	1.77
50	1.55	3.82
51	3.29	—
52	—	—
53	—	2.75
54	—	2.87
55	—	2.04
56	—	2.95
57	—	—
58	—	2.79
59	—	3.81
60	—	3.28
61	—	2.86
62	—	1.48
63	—	11.50
64	—	—
65	—	—

*Dash indicates half-life not calculable

Supplementary Table S10. Post-smoking Δ^9-THCV half-lives in breath

	Half-Life (min)	
	Time Intervals Post-Smoking (min)	
Subject	0-10	0-20
31	—*	—
32	—	—
33	—	—
34	—	—
35	—	3.94
36	—	—
37	—	—
38	—	—
39	—	—
40	—	—
41	6.20	—
42	13.67	—
43	—	—
44	2.09	—
45	2.86	—
46	—	—
47	2.17	—
48	4.40	—
49	1.67	—
50	1.48	—
51	3.32	—
52	—	—
53	—	—
54	—	2.71
55	—	1.96
56	—	2.62
57	—	—
58	—	2.72
59	—	3.38
60	—	—
61	—	—
62	—	—
63	—	—
64	—	—
65	—	—

*Dash indicates half-life not calculable

Supplementary Table S11. Post-smoking CBG half-lives in breath

	CBG Half-Life (min)	
	Time Intervals Post-Smoking (min)	
Subject	0-10	0-20
31	—*	—
32	—	2.74
33	—	6.20
34	—	—
35	—	4.48
36	—	2.66
37	—	—
38	—	—
39	—	—
40	—	—
41	4.50	3.93
42	8.69	—
43	4.90	—
44	2.09	2.41
45	2.61	—
46	4.50	—
47	1.96	2.62
48	4.22	2.96
49	1.79	—
50	1.45	2.56
51	3.43	—
52	—	—
53	—	2.42
54	—	2.69
55	—	1.92
56	—	2.67
57	—	—
58	—	2.80
59	—	3.65
60	—	3.00
61	—	2.39
62	—	1.45
63	—	15.96
64	—	—
65	—	3.66

*Dash indicates half-life not calculable

Supplementary Table S12. Summary of cannabinoid half-lives in breath post-smoking: back-to-back sampling strategy

	Cannabinoid Half-Lives (min) 20 & 40 Minutes Post-Smoking									
	Δ⁹-THC		CBN		CBC		CBG		Δ⁹-THCV	
**Subject**	**20**	**40**	**20**	**40**	**20**	**40**	**20**	**40**	**20**	**40**
66	1.2	—*	1.1	—	1.0	—	0.7	—	0.9	—
67	1.9	2.2	2.0	2.9	2.2	2.3	1.6	—	1.9	—
68	2.3	9.9	2.3	4.8	2.0	3.5	1.3	—	2.1	—
69	2.9	—	2.7	—	2.7	—	2.6	—	2.8	—
70	1.0	3.7	—	—	—	—	—	—	—	—
71	1.4	—†	—	—	1.3	—	—	—	—	—
72	2.1	5.9	1.8	—	2.0	5.2	2.1	—	1.6	—
73	7.3	8.6	6.4	10.7	11.8	—	—	—	3.8	—
74	11.8	—	—	—	—	—	—	—	—	—
**Average=**	3.5	6.1	2.7	6.1	3.3	3.6	1.7	—	2.2	—
**SD=**	3.6	3.2	1.9	4.1	3.8	1.5	0.7	—	1.0	—
**N=**	9	5	6	3	7	3	5	—	6	—

*Half-life not calculable.
†Outlier value (53.1) removed.
